# Analysis of Movement, Orientation and Rotation-Based Sensing for Phone Placement Recognition

**DOI:** 10.3390/s151025474

**Published:** 2015-10-05

**Authors:** Ozlem Durmaz Incel

**Affiliations:** Department of Computer Engineering, Galatasaray University, Ciragan Cad. No:36, Ortakoy, Istanbul 34349, Turkey; E-Mail: odincel@gsu.edu.tr; Tel.: +90-212-2274480 (ext. 307)

**Keywords:** motion sensors, accelerometer, gyroscope, linear acceleration, gravity, magnetometer, mobile phone sensing, phone placement recognition, classification

## Abstract

Phone placement, *i.e.*, where the phone is carried/stored, is an important source of information for context-aware applications. Extracting information from the integrated smart phone sensors, such as motion, light and proximity, is a common technique for phone placement detection. In this paper, the efficiency of an accelerometer-only solution is explored, and it is investigated whether the phone position can be detected with high accuracy by analyzing the movement, orientation and rotation changes. The impact of these changes on the performance is analyzed individually and both in combination to explore which features are more efficient, whether they should be fused and, if yes, how they should be fused. Using three different datasets, collected from 35 people from eight different positions, the performance of different classification algorithms is explored. It is shown that while utilizing only motion information can achieve accuracies around 70%, this ratio increases up to 85% by utilizing information also from orientation and rotation changes. The performance of an accelerometer-only solution is compared to solutions where linear acceleration, gyroscope and magnetic field sensors are used, and it is shown that the accelerometer-only solution performs as well as utilizing other sensing information. Hence, it is not necessary to use extra sensing information where battery power consumption may increase. Additionally, I explore the impact of the performed activities on position recognition and show that the accelerometer-only solution can achieve 80% recognition accuracy with stationary activities where movement data are very limited. Finally, other phone placement problems, such as in-pocket and on-body detections, are also investigated, and higher accuracies, ranging from 88% to 93%, are reported, with an accelerometer-only solution.

## 1. Introduction

Smart phones provide a rich set of context information, not only about the state of the device, but also about the user’s context with the presence of different integrated sensing modalities [1,2,3,4]. Considering the device functionality, inferring where the phone is carried by using sensing information, such as light, proximity and acceleration, can enable a range of context-aware services. For instance, the volume and vibration of the phone can be increased if the phone is detected in an enclosed position, such as a bag or pocket, in order to prevent missing incoming calls and messages [5]. Switching off the screen and the keypad, declining incoming calls, increasing the text size on the screen and providing different authentication schemes are some other examples of context-aware services for improving user experience. Besides such services related to the device functionality, knowing the phone position is also important for mobile phone sensing applications. For instance, an application may want to only take a sound sample for a city-wide noise map when the phone is out of the pocket or bag [6]. Similarly, activity recognition applications running on smart phones can benefit from the phone placement information to improve their accuracies [3].

One of the challenges in identifying phone placement is the wide variety of phone placements adopted by users. In [7], Ichikawa *et al.* interviewed 419 people from three different cities, and in their extended work [5], they conducted a series of street interviews in 11 cities on four continents to identify the main phone carrying options in different cultures. According to the results of this survey, 60% of men reported that they carried the phone in their pocket, while 61% of women reported the bag position. The other common locations were reported to be the belt clip, upper body and hand. However, the use of smart phones has vastly increased since these publications, and motivated by this fact, in a more recent study [8], Wiese *et al.* interviewed 693 people through in-person interviews and Mechanical Turk surveys. Additionally, they collected two weeks of accelerometer data from 32 participants to examine where people keep their phones throughout the day and what factors impact this decision. Compared to the results of the previous surveys, the most common location reported by 68% of the participants is “out on table” or “desk” considering the 24-h usage. The other most popular places are reported to be front trouser pocket (13%), purse (4%), bag/backpack (2%), hand (2%), back trouser pocket (1%) and case (1%). They observe a similar gender difference in phone placement around trouser pockets and purses as the previous surveys [5,7]. A significantly higher percentage of men (20%) report carrying the phone in their front trouser pocket, whereas all 27 participants who reported to have the phone in their purse were reported to be the female participants. Additionally, it was reported that the placement of the phone changes according to the user’s activity/location, such as walking, driving, at home and at the office.

Motivated by these facts, in this paper, the phone placement/position recognition problem using only the accelerometer is analyzed, by focusing on the following question: “How much does the phone move, change its orientation and rotation at different positions and how can this information be extracted from acceleration readings and used in identifying the phone positions?”. This question is motivated by the study in [9]. Kunze *et al.* mention that “when motions are dominated by rotations, we should avoid acceleration features; gyroscopes provide information that is invariant to body part displacement”. However, instead of using the gyroscope for rotation information, we extract rotation-related features, namely pitch and roll, from the acceleration readings and investigate how motion, orientation and rotation changes improve the recognition accuracy. Three different datasets [10,11,12,13], which were primarily collected for position-independent activity recognition, are utilized. In these datasets, 35 subjects participated in the data collection phase, and the accelerometer was the common sampled sensor, whereas in [11] and in [12], linear acceleration and gravity readings were also collected. Additionally, gyroscope and magnetic field sensors were also sampled in [12]. The idea is that many datasets are collected for the position recognition problem; however, they are analyzed in isolation, and they are usually limited in terms of the number of participants and positions. My idea is to create a pool of training data collected from different positions and to analyze the scalability of the classification algorithms to the new positions.

Features from the magnitude of acceleration are used as the motion-related features, whereas features from the individual axes of the accelerometer are utilized to compute the orientation-related features. Additionally, features from the pitch and roll values are utilized as the rotation-related features. Following a systematic approach, it is shown how the recognition performance can be increased with the use of orientation and rotation-related features besides the motion-related features using only the accelerometer in the first set of tests. In the second round of tests, it is investigated whether an accelerometer-only solution can achieve the same rate of accuracies with the use of linear acceleration, gravity, gyroscope and magnetic field values. In the third set of experiments, instead of focusing on the exact phone position identification, I focus on in-pocket detection and on-body detection problems by aggregating the original positions into a reduced number of positions. Finally, the impact of the activity performed by the subjects, such as walking, stationary and mobile, on the position recognition performance is investigated. It is shown that if the activity can be identified before, then the accuracies generally increase. In the tests, the performance with four different classifiers was evaluated. To summarize, I analyze the impact of using different feature sets, using different sensors and classification algorithms on the performance of the phone placement problem considering different positions, different activities performed by subjects and different placement problems. The main contributions and highlights of this paper are summarized as follows:

- The performance of phone placement detection using motion, orientation and rotation information from acceleration-only signals is investigated. The accelerometer-only solution is preferred since it is one of the least power-consuming sensors, and it is shown that the accelerometer performs with 76% accuracy for Dataset 1 [11], which is the most challenging dataset due to two pocket and two bag positions, with 93% accuracy for Dataset 2 [12], with 88% accuracy for Dataset 3 [13] and with 85% accuracy when the datasets are combined into eight different positions.
- It is shown that using only motion, orientation or rotation information alone does not perform well. However, when motion information is combined with rotation information, accuracies increase for most of the positions, and using three modalities together often increases the accuracies. Additionally, a random forest classifier is observed to provide higher accuracies compared to other algorithms.
- Besides the acceleration information, the use of gyroscope and magnetic field information for phone position identification is also investigated. It is observed that the gyroscope and magnetic field sensors alone do not perform as well as the accelerometer. However, when the gyroscope is utilized for rotation information, it exceeds the accuracy achieved with only the acceleration solution, though the increase was not remarkably high: only 2%.
- When different placement problems are considered, accuracies increase by 4% to 7% for in-pocket detection and on-body detection, respectively, compared to exact placement recognition.
- It is investigated whether the activity being performed may change the signal behavior of a position, particularly considering motion and orientation sensors and observing that walking activity often makes it easier to detect the positions. It is also shown that an accelerometer-only solution can achieve 80% recognition accuracy even with stationary activities where movement data are very limited.

The rest of the paper is organized as follows. In Section 2, the related work on phone placement detection using various sensor information is presented. Section 3 introduces the methodology for phone placement detection, the utilized datasets, the usage of motion and orientation information, the list of features and the details of the classifiers. In Section 4, first, the experimental setup is explained, and then, the results of the applied methods are elaborated. In Section 5, a discussion of the achieved results is presented where the findings are elaborated, and it is discussed how they can be useful in future studies or in practice. Section 6 finally draws the conclusions.

## 2. Related Work

The problem of sensor/phone placement identification for different application areas, particularly context-aware applications, has been studied previously with the use of different sensing modalities. In their early work, Kunze *et al.* [14] highlighted the importance of the locations of wearable sensors, and in their recent work [9], they explore how the sensor placement variations impact the results of human action recognition.

In some of the previous studies, the solution is based on using different types of sensor modalities. For example, in [15], preliminary results on differentiating between in-pocket *versus* out-of-pocket positions were presented. In this study, the microphone was used as the sensing modality, and 80% accuracy was reported to differentiate between these two states. In-pocket detection was also studied in [16] using light and proximity sensors. Similarly, in the Sensay study [17], the light sensor was used for the in-pocket detection. In this paper, the recognition of in-pocket *versus* other positions is also investigated, and using the acceleration information, an accuracy of 93% detection performance is achieved with a relatively higher number of other states compared to these studies.

The use of only acceleration information for the recognition of the placement sites is explored in recent studies [9,18], however, rather than phones, wearable sensors were used in the data collection. In [19], the focus was again on using accelerometer data for position recognition, and similarly, an external sensor box (Nokia Sensorbox) was used in the data collection. Positions were identified while the subjects were walking, and the target classes were bag, ear, hand and pocket. The average recognition accuracy was presented as 94%. Compared to this study, in this paper, a larger set of phone positions is targeted, and different sets of activities were performed by the subjects, besides the walking activity. In another study [20], only the acceleration sensor was utilized, targeting nine positions, four different pocket positions, four different bag positions and the around the neck position. Twenty seven features from individual axes were extracted from the acceleration readings. Compared to this study, positions other than the bag and pocket positions are targeted, such as hand, belt and upper arm, and also, I investigate the use of other sensing information from motion sensors, not only focusing on walking activity; the dataset also includes readings from stationary and other mobile activities.

In [21], a rotation-based approach was proposed using accelerometer and gyroscope readings. The rotation radius and angular velocity were used as the primary features. Using the SVM classifier with cross-validation approach, 91% accuracy was achieved with four phone positions. Four subjects participated in the study, and they were walking during the data collection phase. In this study, the use of rotation-based features is also investigated besides the motion and orientation-related features in a bottom-up approach with a higher number of participants, activities and positions. Moreover, an accelerometer-only solution is proposed for computing rotation-related information instead of utilizing a gyroscope.

In [22], the impact of sensor positions on sensor values is investigated, including the accelerometer, microphone, gyroscope, GPS, magnetometer and light sensor. Additionally, a position discovery service is presented using the accelerometer and gyroscope sensors. In this solution, first, the activity of the subject is identified, such as still, walking and running, and the on-body position is identified according to the activity. Ten participants were included in the data collection phase, and six positions were targeted: three hand positions, holding, watching a video and talking on the phone, and two pocket positions, hip pocket and jacket pocket. Eighty eight percent accuracy was reported when the accelerometer is used, and 89% accuracy was reported when both sensors are used together. The positions targeted in my study are quite similar to this study. However, in this paper, the combined dataset is more challenging, including 35 participants, a higher number of classes (eight positions), including different sets of activities, and the classification accuracies are reported using a leave-one-subject-out method without the requirement of user-specific training data.

In [8], the results of a comprehensive survey on the common phone positions is presented, and a multi-spectral material sensor, which is external to the phone, is utilized, besides the accelerometer, light/proximity and capacitative sensors. Pocket, bag, hand and out positions were considered, and 85% accuracy was achieved. The use of the microphone together with the accelerometer was also studied in [23]. Three positions (hand, pocket and backpack) were targeted; 84% accuracy was achieved with the accelerometer using the random forest classifier, and 89% accuracy was achieved with the use of the microphone. When the two sensing modalities are combined, the accuracy was reported to be 93%. However, the use of the microphone may not be practical and energy efficient for continuous phone position recognition.

In another recent work, Wahl *et al.* [24] focused on the use of RFID-based tagging for phone position recognition. Instead of using an expert-based approach for labeling at the data collection phase, RFID tags are used. In their extended work [25], it is shown that, RFID-based tagging, performs only 2% below the expert-verified variant of labeling, which is costly and not practical to obtain in daily life studies. The highest accuracy was reported as 84% in terms of normalized accuracy, where the accelerometer, light and proximity sensors are utilized with five classes, pants, table, jacket, bag and other. In Section 5, I present a detailed comparison with existing acceleration-only solutions in the literature.

The datasets that are utilized in this paper were presented in earlier work [11,12,13]. In these studies, the focus was on position-free activity recognition, and for this purpose, activity data from different positions were collected. In [13], the phone placement recognition problem using the accelerometer data was partially addressed where the main focus was on investigating how much increase in activity classification can be achieved with phone position information compared to position-independent activity recognition. The use of pitch and roll values besides the features from the magnitude of acceleration was studied. However, the detailed analysis of motion and rotation-related features were not explored; orientation features were not utilized, and the fusion of such features was not investigated. A limited number of features were extracted; only the random forest classifier was used, and the findings were only applicable to this dataset. Moreover, the other phone placement problems, such as in-pocket detection, that I analyze in this paper were not targeted, and the impact of activity data on position recognition was not analyzed. In [12], gyroscope and magnetometer data were also collected aiming at the investigation of sensor fusion for better activity recognition performance. In [11], position, orientation and device model independent activity recognition was targeted. Some preliminary results on the phone position classification were presented using the same set of features used for activity recognition. Around 71.5% accuracy is achieved for position recognition with the KNN classifier and 69% for the decision tree classifier. In this paper, on the other hand, a systematic approach is followed to evaluate the motion, orientation and rotation-related features extracted from the accelerometer readings. It is also investigated how the accelerometer alone performs compared to the use of gyroscope, magnetic field, linear acceleration and gravity sensors. Additionally, other position recognition problems, such as on-body detection, and enclosed-position detection are investigated with the use of accelerometer data.

## 3. Methodology for Phone Placement Recognition and Datasets

In this section, the methodology that is followed for the recognition of phone positions is explained, as well as the characteristics of the datasets utilized in this study. All of the datasets [11,12,13] include raw sensing information together with the timestamp of the sample, as well as the position tag. First, the samples are segmented into different time windows, and different features from this segmented data are computed. Finally, different classifiers are trained, and classification tests based on a leave-one-subject-out method are performed.

### 3.1. Characteristics of the Datasets

First, I explain the details of the three datasets utilized in the evaluations. The requirement for the dataset selection was that it should be collected with motion sensors, particularly with the accelerometer, integrated on a smart phone, since the focus of the paper is on the efficiency of an only-acceleration solution. Another requirement was that the data should be collected simultaneously from different positions to analyze the variances in signals from different positions. Additionally, the other requirement was that it should be collected when participants perform different sets of activities, not focusing on a single activity, such as walking, and should include both stationary and mobile activities. In this respect, the datasets that could be considered are from [8,20,22,25,26,27]. However, in [20], only the walking activity was considered, and in [8,25], the data collection was not simultaneous with multiple phones located in different positions, but the data from different positions was collected at different times. The other datasets [22,26,27] could not be used, since they were not publicly available for download, such as [22,26], or agreements for data sharing were not completed during the manuscript preparation, particularly for the dataset in [27]. However, I plan to include the dataset from [27] once the agreement has been completed, in future work. Particularly, three different datasets are utilized, which were collected in previous work [10,11,12,13]. A summary of the characteristics of these datasets is presented in Table 1.

**Table 1:** Characteristics of the utilized datasets.

	Dataset 1 [11]	Dataset 2 [12]	Dataset 3 [13]
**Positions**	4: Backpack, Messenger Bag, Jacket Pocket, Trouser Pocket	5: Trouser Pocket, Upper Arm, Belt, Wrist	3: Backpack, Hand, Trouser Pocket
**Activities**	5: Sit, Stand, Bike, Walk, Run	6: Walk, Stand, Jog, Bike, Stairs, Sit	9: Sit, Stand, Walk, Run, Transport, Stairs, Secondary Activities: sending an SMS, making a call, interaction with an app
**Sensing Modalities**	Acceleration, Linear Acceleration, Gravity	Acceleration, Linear Acceleration, Gravity, Gyroscope, Magnetic Field	Acceleration
**Sensor Sampling rate**	100 Hz	50 Hz	100 Hz
**Phone Models**	Samsung Galaxy S3	Samsung Galaxy S2	Samsung Galaxy S2 and S3 Mini
**No. of Subjects**	10	10	15

In [11], acceleration data, gravity, linear acceleration data in phone coordinates and linear acceleration data in Earth coordinates were collected from 10 participants at 4 different positions: backpack, messenger bag, trousers’ pocket and jacket pocket, from 4 phones simultaneously. Users performed 5 different activities, walking, running, biking, standing and sitting, where each activity was performed for approximately 3 min. In total, 600 min (15 min per participant, 4 phones) of data were collected. Except the pocket positions, in other positions, although the phone is carried by the user, it indirectly captures the user movements, since it was worn by the user, but not directly attached to the body.

In [12], data from acceleration, linear acceleration, gyroscope and magnetic field sensors was collected. Ten participants performed similar activities, such as walking, jogging, stairs (up/down), biking, sitting and standing, while the phones were attached to the upper arm, wrist, jean pocket (right and left) and belt positions. In total, approximately 840 min of data (21 min of data for each participant, 4 phones) are available in this set. While the pocket and belt positions are commonly used for carrying the phone, the arm position is usually used when training activities, like jogging, are performed. The wrist position is used to simulate the position of a smart watch. In this dataset, all phones could capture user movements, since they were directly attached to the user body.

In [13], acceleration data from 15 participants at three different positions, hand, backpack and pocket, were collected. In this dataset, similarly, participants were walking, running, standing, going up/down stairs, sitting and transported by a bus following a predefined scenario. Additionally, there were secondary activities, such as making a phone call, interacting with the phone and sending an SMS, while performing the stationary activities to make the data collection more realistic and closer to the daily usage of the phone. In total, approximately, 900 min of data (around 20 min of data per subject, 3 phones) were collected. Except the backpack position, in hand and pocket positions, the phone could directly capture the user movements, since it was attached to the body.

In general, as presented in Table 1, all of these sets include information where the phone was attached to the body, such as the arm and wrist, or carried/worn by the user, such as the jacket pocket, backpack and messenger bag. The trouser/jean pocket was the common position in all 3 sets, and although it is a pocket position, since it captures the movement/orientation of the leg, it can be considered as attached to the body.

While collecting the data, activity and position information was also recorded as the ground truth data in all three sets. In Dataset 1 [11], an interface was available on the phone screen, and before placing the phone into a specific position, the user was selecting the position from the interface. The first and last couple of seconds of data were cropped to remove the data from placement action. In Dataset 2 [12], similarly, the data collection app running on the phone had an activity labeling interface, and each activity was labeled through this interface. In Dataset 3 [13], there was an external observer who was carrying another phone. The observer used the phone as a chronometer and labeled the start and stop times of the activities. Before the data collection, the clocks of the phones were synchronized: all of the phones were put on a flat surface, and a strong knock was applied to the surface. According to the timing differences of this hit recorded on the phones, clocks were synchronized while labeling the data.

In my performance evaluations in Section 4, experiments with individual datasets are performed, as well as the combination of datasets. Overall, the combined dataset includes data from 35 subjects from 8 different phone locations, and 8 different activities were performed by the subjects, which makes it a much larger and more complex dataset, compared to similar studies in the literature.

### 3.2. Sensing Modalities

As mentioned, all of the datasets include raw acceleration readings, whereas the first [11] and second datasets [12] include linear acceleration and gravity information, whereas the second set includes also gyroscope and magnetic field sensor information.

The integrated accelerometer on Android phones measures the gravitational acceleration, if the device is stationary or its speed does not change. If the phone is accelerating, it measures the combination of the gravitational acceleration and the acceleration due to movement, in m/s${}^{2}$. This acceleration due to movement, other than the gravitational force is named the “linear acceleration”. Both the raw acceleration and the linear acceleration are measured in 3 axes, *x*, *y* and *z*, in terms of phone coordinates. When a smartphone is put on a horizontal surface, the *x* axis is horizontal and points to the right, the *y* axis is vertical and points up and the *z* axis points from the screen. This coordinate system is independent from the orientation of the phone. The same coordinate system is used for gravity, gyroscope and magnetic field sensors.

The gravity sensor on the Android platform provides a three-dimensional vector indicating the direction and magnitude of gravity, and the unit is the same as the acceleration sensor, m/s${}^{2}$. The gyroscope sensor measures the rate or rotation in rad/s around the *x*, *y* and *z* axes of the phone. The geomagnetic field sensor, *i.e.*, magnetometer, monitors changes in the Earth’s magnetic field in μT.

No pre-processing algorithm was applied on any of the datasets to remove any possible noise. As I discuss in Section 3.3, frequency domain features are extracted besides time domain features, and frequency parameters can help to avoid some of the noise added while collecting data.

### 3.3. Feature Extraction and Selection

One of the main themes of this paper is the evaluation of classification performance with different feature sets, namely the motion-related features, orientation-related features and rotation-related features. The raw acceleration readings include both the dynamic (due to movement of the phone) and static acceleration (due to gravity) values, and it is not possible to separate them when the phone is moving without using gravity readings. However, in my study, it is not necessary to compute the exact orientation of the phone. Instead, we are focusing on the changes in the acceleration readings in the individual axes. Hence, I use the magnitude of acceleration for the computation of motion-related features; whereas I use the readings from each of the 3 axes for the computation of orientation-related features. One may argue that the features that I consider as “orientation-related features” are also motion-related features in individual axes, since they are measured when the phone was moving (except the static activities). However, as mentioned, due to the gravity component in the raw acceleration, the changes in the readings on different axes are different. Similarly, considering the rotation-related features, the exact pitch and roll angles are not computed in the analysis, but changes in the computed values are calculated and used as features.

In [22], it was shown that the magnitude of acceleration (square-root of the sum of the squares of readings in each accelerometer axis) exhibits different behavior for different phone positions. From the raw acceleration readings, the following motion-related features are computed from the magnitude values computed over a time-window:

- Mean: the average value of the magnitude samples over a time window.
- Variance: the average of the squared differences of the sample values from the mean value over a time window.
- Root mean square (RMS): the root mean square is the square root of the sums of each datum over a window, divided by the sample size.
- Zero-crossing rate (ZCR): the number of points where a signal crosses through a specific value corresponding to half of the signal range; in our case, the mean of a window is utilized.
- Absolute difference (ABSDIFF): the sum of the differences from between each magnitude sample and the mean of that window divided by the number of data points. This feature was utilized in [22] for individual acceleration axes to enhance the resolution in capturing the information captured by data points.
- First 5-FFT coefficients: the first 5 of the fast-Fourier transform coefficients are taken since they capture the main frequency components, and the use of additional coefficients did not improve the accuracies.
- Spectral energy: the squared sum of spectral coefficients divided by the number of samples in a window.

Orientation changes of the phone can be calculated from the accelerometer readings from each axis, since they include gravitational acceleration values. As the orientation-related features, the following features are extracted from each accelerometer axis, resulting in a total of 12 features:

- Standard deviation: square root of variance.
- Root mean square (RMS)
- Zero-crossing rate (ZCR)
- Absolute difference (ABSDIFF)

Although the orientation of the phone may not change, the phone may be subject to rotational changes, and this information can be different in different positions. The rotational information can be extracted from the gyroscope or orientation sensor on Android phones; however, this requires the use of other sensors, and the orientation sensor was deprecated in Android 2.2 (API Level 8). In my previous work [13], I extracted pitch and roll information from the acceleration readings. Equation (1) was used for computing the pitch value and Equation (2) was used for computing the roll value. *x*, *y* and *z* represent the accelerometer values in the associated coordinates, whereas *g* is the gravitational acceleration, *i.e.*, 9.81 m/s${}^{2}$, (atan2, the arctangent function with two arguments is used in computing these equations):

(1) $$\text{β}=\frac{180}{\Pi }.tan^{-1}\left(y/g,z/g\right)$$

(2) $$\text{α}=\frac{180}{\Pi }.tan^{-1}\left(x/g,z/g\right)$$

After calculating the pitch and roll values, the following rotation-related features are extracted for both pitch and roll values, resulting in 12 more features:

- Mean
- Standard deviation
- Root mean square (RMS)
- Zero-crossing rate (ZCR)
- Absolute difference (ABSDIFF)
- Spectral energy

In total, 35 features are extracted from the accelerometer readings in each dataset. Different sets of these features were used in previous studies [8,22]. Here, it is aimed to explore their performance over different datasets. Feature selection using the WEKA machine learning toolkit’s [28,29] (Waikato Environment for Knowledge Analysis), feature selection methods, such as CFSSubsetEval and InfoGain, are also applied. However, the overall results with the entire set of features were observed to be higher.

**Figure 1:**
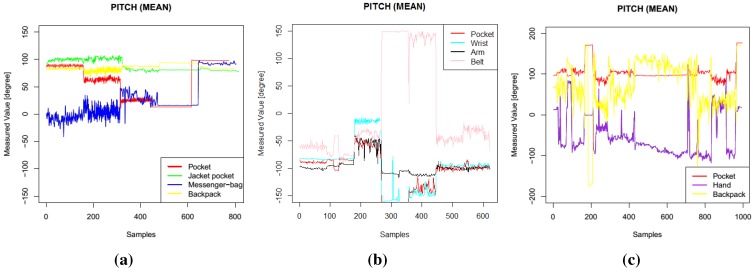
Pitch features. (**a**) Dataset 1; (**b**) Dataset 2; (**c**) Dataset 3.

**Figure 2:**
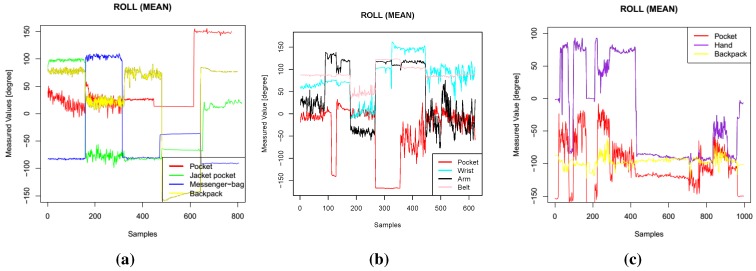
Roll features. (**a**) Dataset 1; (**b**) Dataset 2; (**c**) Dataset 3.

**Figure 3:**
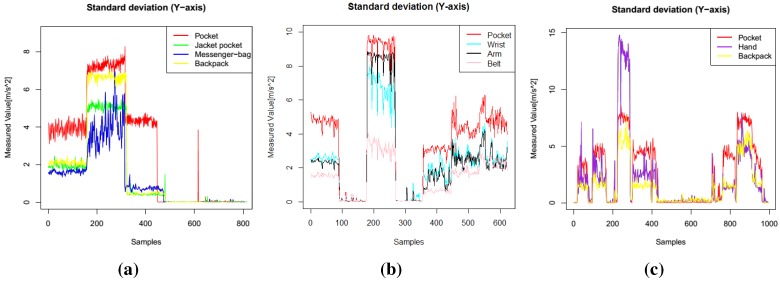
Standard deviation, *y* axis features. (**a**) Dataset 1; (**b**) Dataset 2; (**c**) Dataset 3.

Example plots of different features (pitch, roll, standard deviation of *y* axis) are presented in Figure 1, Figure 2 and Figure 3. In these figures, data from one participant is presented. The sequence of activities was as follows for Dataset 1: walk, run, bike, sit, stand; for Dataset 2: walk, stand, jog, sit, bike, upstairs, downstairs; for Dataset 3: stand, walk, stairs, stand, stairs, walking, stairs, walk, sit, stand, walk, stand, run, stand, walk, stand, transportation, walk, sit. These three features were among the most selected features when I applied the feature selection algorithms. In Dataset 1, the mean pitch values are similar while the participants were performing stationary activities, such as sit and stand (the last 320 sequences), as shown in Figure 1a. However, the roll values are quite different for these activities, as presented in Figure 2a. Similarly, the standard deviation values in the *y* axis, as shown in Figure 3, show different patterns during mobile activities, such as running and walking, for the three datasets.

Linear acceleration and gravity readings provided by the Android API were also recorded in Dataset 2 [12] and Dataset 3 [13]. Similar to raw acceleration readings, the following features are extracted from linear acceleration readings: mean, variance, RMS, ZCR, ABSDIFF, first five FFT coefficients and spectral energy features from the magnitude of linear acceleration. From the gravity information, standard deviation, RMS, ZCR and ABSDIFF values from each of the axes and the pitch and roll features (mean, standard deviation, RMS, ZCR, ABSDIFF, spectral energy) were extracted, as well.

Gyroscope and magnetic field sensor readings provided by the Android API were available in Dataset 3 [12]. Standard deviation, RMS, ZCR and ABSDIFF features from each of the axes were extracted. In Section 4.3, I evaluate the recognition performance with these sensors alone and together with acceleration readings.

### 3.4. Classification

In the classification phase, four classification algorithms are used: K-nearest neighbor (KNN), decision tree, random forest and multi-layer perceptron. By using a similar set of algorithms used in previous studies [8,20,22,23], my aim is to provide comparable results.

KNN is an instance-based learning algorithm. Given *N* training vectors, the KNN algorithm identifies the K-nearest neighbors of any test sample vector using a distance metric, such as the Euclidean distance or Mahalanobis distance. In my analysis, the IB1 version of KNN on the WEKA platform [28,29] is used with the Euclidean distance, and the K value is equal to 1.

The decision tree algorithm builds a classification tree based on the given observations and by splitting training data into subsets based on the feature values. The target classes are positioned at the leaves of the tree. In my analysis, the J48 version of the decision tree algorithm available on the WEKA platform was used.

The random forest algorithm utilizes multiple decision trees and constructs a forest of decision trees at the training phase. In the classification phase, the class that appears most often in the constructed trees is selected based on a majority voting mechanism. In my analysis, the WEKA version of the classifier with ten trees constructed at the training phase is used.

Multi-layer perceptron (MLP) uses a feed-forward artificial neural network model, consisting of multiple layers of nodes. MLP utilizes a supervised learning technique called back-propagation for training the network. In this paper, the WEKA version of MLP with the default settings is used.

In the classification phase, the leave-one-subject-out (LOSO) approach was used. In the test phase, data from one person are used as the test data, while the data from all other participants were used as the training data. For instance, when the datasets are combined (results are reported in Section 4.1), the average value (the computed averages were the weighted averages provided by the WEKA platform, taking into account the number of instances for each person and class) calculated from 35 (35 participants in total) tests is reported. Using the LOSO approach, a more realistic situation can be represented. It may be the case that a person may not have any data collected from a specific position, and in this way, the scalability of the approach to the new users and placements can be measured.

## 4. Performance Evaluation

In this section, the results of the evaluations following the methodology explained in Section 3 are explained. Before the classification, in the preprocessing phase, the R programming environment [30] is used for feature extraction. The three datasets include only raw sensor information, and initially, the same set of features is extracted. Next, in the feature selection and classification phases, the WEKA machine learning toolkit [28] is utilized. The WEKA platform provides a number of different classification and feature selection algorithms.

For performance evaluation, the parameters that may affect the performance of the position recognition and the metrics to report the performance are identified:

- Phone-position: This is the target class that I aim to identify in the recognition process. For phone position, initially, exact phone positions are targeted, which include backpack, messenger bag, trouser pocket, jacket pocket, hand, belt, arm and wrist. Moreover, the recognition performance is also evaluated when the number of classes is reduced, since knowing the exact position may not be necessary. In this case, the original classes are aggregated as pocket, bag, upper-body, lower-body, hand or other. The impact of aggregating such classes in the recognition performance is analyzed.
- Sensors: The three datasets that are utilized in this paper all include raw accelerometer information, whereas two sets [11,12] include raw linear acceleration and gravity values. Additionally, the third dataset [12] includes the raw gyroscope and magnetic field sensor readings. The impact of using different sets of sensors on the performance is analyzed.
- Features: Features are the summaries or, in other words, the signatures of the raw sensing information. The impact of using orientation-free features, such as magnitude of acceleration, using orientation-dependent features, reading in the individual axes of the sensors, as well as the rotational features, such as the pitch and roll values, is analyzed.
- Classification algorithms: I applied different classification algorithms for the evaluation of recognition performance. The selected classifiers include, decision trees, random forests, multi-layer perceptron and KNN classifiers. These classifiers have been used in the state-of-the-art studies, and this makes it easier to compare the findings to the related studies. The classification was performed on a subject basis using the leave-one-subject-out (LOSO) method where all individuals were evaluated separately.
- Activities: Since we are dealing with the recognition problem using motion and orientation sensors, the activities performed by the users in the datasets play an important role for the sensor readings. In some of the previous studies, either position recognition with one single activity was proposed, such as walking [19,21], or first, the activity was recognized and then position classification was performed [18,22]. In order to evaluate the impact of the activity, I also carried out experiments where the sensor readings with all of the activities, with only the walking activity, with stationary activities (sitting, standing) and with mobile activities (running, walking, stairs, transportation, biking) were available.

In my methodology, features are extracted over a number of sensor readings instead of individual readings. Initially, the impact of using different window sizes, *i.e.*, time intervals, on the recognition performance is investigated using 1 s, 2 s and 4 s of %50 overlapping windows. The results with the 2-s windows were slightly higher compared to other sizes; hence, I continued with the 2-s window size. As the performance metrics, the “accuracy (true-positive rate)” and the “F-measure”, values ranging between zero and one, are used. Accuracy is defined as the ratio of correctly-classified examples of a specific class over all of the examples of the same class, as provided in Equation (3). F-measure calculation is based on precision and recall values, which is provided in Equation (4). When the averages are computed for different positions, weighted averages are presented where the number of instances for each specific position is taken into account.

(3) $$\begin{array}{c} Accuracy=\frac{\sum_{i=1}^{N}TP_{i}}{Total} \end{array}$$

(4) $$\begin{array}{c} F-Measure=\frac{2\times precision\times recall}{precision+recall} \end{array}$$

### 4.1. Placement Detection with the Accelerometer

**Figure 4:**
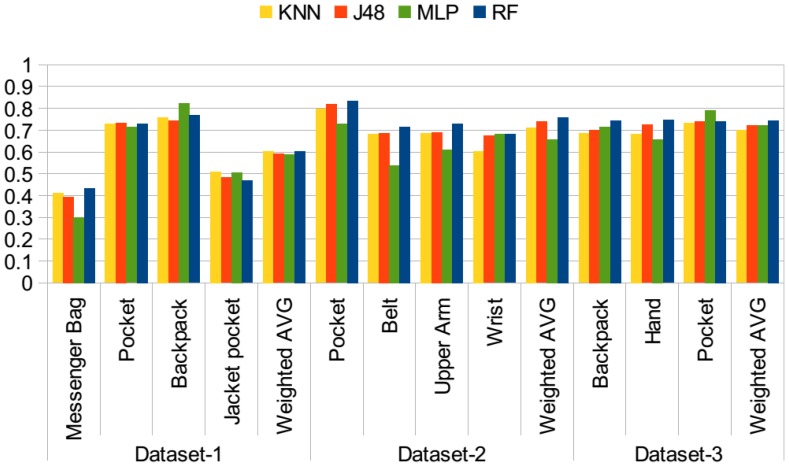
Position recognition performance with only motion features.

As mentioned, the accelerometer was the common sensor used in all three datasets. In the first set of experiments, the use of only motion-related information, namely the mean, variance, RMS, ZCR, ABSDIFF, first five FFT coefficients and spectral energy of the accelerometer magnitude, which were explained in Section 3, is investigated. The idea is that, if the phone is attached to the body, the acceleration would be lower, whereas if it is in a bag or a pocket, it may move freely. It was also visualized in [22] that the magnitude of the accelerometer exhibits different behaviors on different parts of the body while the same activity is performed. In Figure 4, the results of the first set of experiments using different classifiers are presented. In this test, datasets are examined individually and not combined. The *y* axis represents the accuracy values. Although the accuracy values range between zero and one, in the text, I mention the accuracies in terms of percentages, for the ease of reading. Using only motion-related information performs poorly, since different activities performed by the participants impact the results, as well. In particular, the accuracy results achieved with the first dataset are around 60%, even lower for messenger bag and jacket pocket positions. When the confusion matrices for these two positions are analyzed, it is observed that the jacket position is mostly confused with the messenger bag position, while the messenger bag position is often confused with the jacket pocket and also the other bag position, the backpack. As the examples, the confusion matrices obtained with the random forest classifier for all three datasets are presented in Table 2, Table 3 and Table 4. The values are given in percentages. In Dataset 2, the belt and arm positions exhibit similar accuracies, whereas the pocket position has 11% higher accuracy and wrist position 3% lower accuracy, which is confused with arm and pocket positions. In this dataset, data from right and left pockets were obtained; however, in my analysis, these two were combined into a single pocket position. In Dataset 3, accuracies for the classes are similar, around 74%. As mentioned in previous studies [8], the bag positions are difficult to recognize. In the second dataset, there is no bag position, whereas in the third dataset, only the backpack position is available. These two sets include positions related to different parts of the body; hence, they are easier to differentiate.

**Table 2:** Confusion matrices with motion-related features, Dataset 1 (random forest).

	Messenger Bag	Pocket	Backpack	Jacket Pocket
Messenger bag	**43.48**	12.21	18.30	26.02
Pocket	14.62	**73.11**	6.16	6.11
Backpack	15.56	3.43	**76.84**	4.17
Jacket pocket	36.42	8.05	8.58	**46.94**

**Table 3:** Confusion matrices with motion-related features, Dataset 2 (random forest).

	Upper Arm	Belt	Pocket	Wrist
Upper arm	**72.97**	8.15	8.34	10.53
Belt	11.96	**71.54**	11.50	5.00
Pocket	5.58	5.60	**83.45**	5.37
Wrist	11.01	9.28	11.48	**68.23**

**Table 4:** Confusion matrices with motion-related features, Dataset 3 (random forest).

	Backpack	Hand	Pocket
Backpack	**74.48**	13.68	11.84
Hand	17.37	**74.56**	8.07
Pocket	13.82	12.05	**74.13**

When we look at the performance of different classifiers, on average, they perform similarly, with the exception of the MLP classifier. It performs with lower accuracy for the second dataset, which is due to the belt position, while it exhibits a similar average accuracy in the other two datasets.

**Figure 5:**
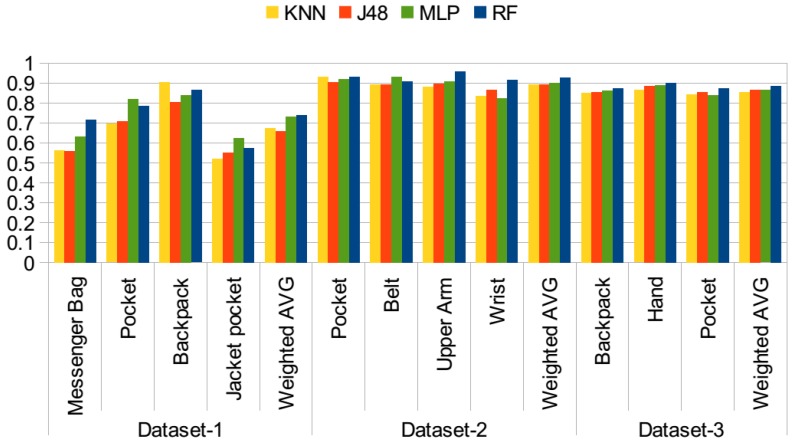
Position recognition performance with motion and orientation features.

In the second set of experiments, the orientation-related features are added. The motivation was again to detect the orientation changes in different positions. This list of features was explained in Section 3. In total, 12 more features are added to the original list of only motion-related features. The results of the experiment are presented in Figure 5. With the addition of these features, in Dataset 1, the average accuracy increased by 13.5% with the random forest classifier, by 7% with the J48 classifier, by 7% with KNN and by 14% with MLP. With Dataset 2, this increase ranged from 15% with the J48 classifier to 24% with MLP. For the third dataset, a similarly remarkable increase is observed for all classifiers, 14% to 15% on average. When the trees constructed by the J48 classifier are analyzed, we observe that the standard deviation in all axes, the RMS values for each axis and the ABSDIFF features are dominant features in identifying these positions.

**Figure 6:**
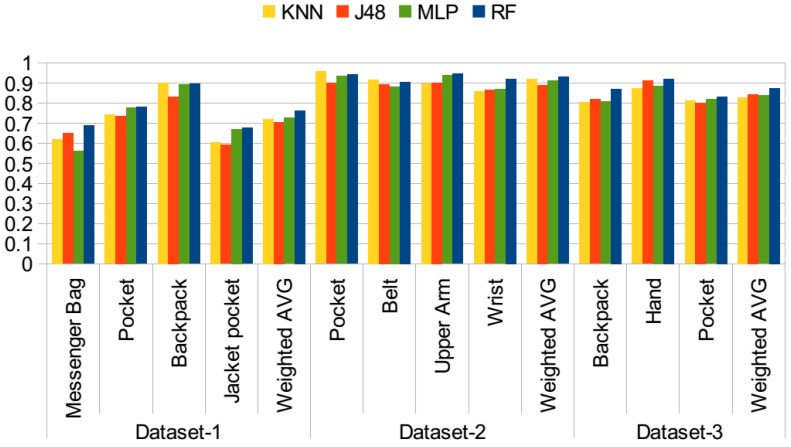
Position recognition performance with all features.

In the third set of experiments, the rotation-related features are also added. As stated before, in previous work [9], Kunze *et al.* mentioned that “when motions are dominated by rotations, we should avoid acceleration features; gyroscopes provide information that is invariant to body part displacement”. Rotational features can be extracted from the gyroscope. However, this requires the use of an additional sensor. In this set of experiments, features from pitch and roll values are extracted as explained in Section 3. The results are presented in Figure 6. Compared to the results achieved with the use of motion and orientation features, given in Figure 5, in Dataset 1, the average ratio increased by 2% with the random forest classifier, whereas the increase was 4% by the J48 and KNN classifiers, and it stayed the same with the MLP classifier. In Dataset 2, average accuracy was differing at most by 2% with KNN, whereas the random forest, J48 and MLP results are the same. In the third dataset, again, similar performance was observed for the random forest classifier, whereas the results of J48, KNN and MLP decreased by 2%. In Dataset 2 and Dataset 3, the accuracies were quite high with motion and orientation-related features, around 90%; however, for Dataset 1, there was room for improvement, and rotation-related features help to separate especially messenger bag and jacket pocket positions better. This is also shown in Table 5 compared to the results obtained with only motion-related features given in Table 2.

**Figure 7:**
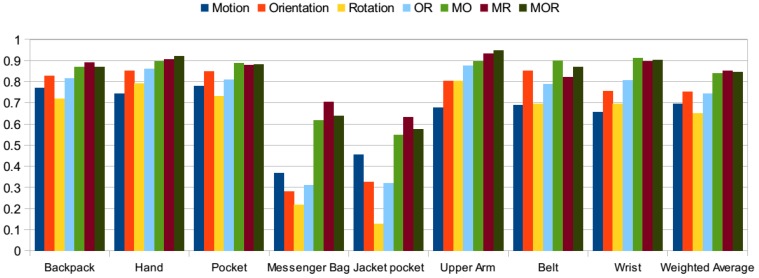
Different feature combinations (M: motion; O: orientation; R: rotation).

The first sets of the experiments were performed on a dataset basis. In the next phase, all of the datasets are combined, achieving a dataset of 35 participants. Results are presented in Figure 7 with different combinations of features using the random forest classifier. As I discussed for the individual datasets, adding rotation-related features besides motion and orientation-related features did not always improve the results. In this test, the aim is to analyze different combinations of features on the combined set in detail. The *y* axis represents the accuracy values.

**Table 5:** Confusion matrices with all features, Dataset 1 (random forest).

	Messenger Bag	Pocket	Backpack	Jacket Pocket
Messenger bag	**69.36**	9.23	9.63	11.78
Pocket	12.76	**76.92**	3.41	6.91
Backpack	4.98	1.32	**89.05**	4.64
Jacket pocket	23.73	5.37	3.59	**67.31**

When the results of individual sets of features are analyzed (only motion, only orientation, only rotation), using only orientation-related features achieves the highest accuracy in most of the positions, except the messenger bag and jacket pocket positions. In these positions, motion-related features achieve higher accuracies compared to only-orientation and only-rotation-related features. Because, the messenger bag position is confused with the pocket and jacket pocket positions, and the jacket position is confused with the messenger bag position. The confusion rate is higher with using only orientation and rotation features, since these positions exhibit similar behavior; they are not strictly attached to the body.

If we analyze the results obtained with the couples of feature sets, such as motion and orientation (MO), motion and rotation (MR) and orientation and rotation (OR), the orientation rotation (OR) set achieves the lowest results, since motion-related features are more dominant in separating the positions given that most of the activities performed by the participants were mobile activities, but the stationary activities, such as sit and stand, were less frequent in the datasets. In most of the cases, motion and rotation-related features (MR) achieve the highest accuracies, especially for messenger bag and jacket pocket positions. When all three sets of features are used altogether (MOR), accuracies are often the same as the motion and rotation-related (MR) features or, in some cases, higher. However, for lower scoring positions, such as the messenger bag and jacket positions, using orientation-related features besides the motion and rotation-related features did not improve the accuracies, but lowered them. Since in these positions, the phone was not tightly placed, but was moving and rotating freely during the activities, the use of this combination improved the results.

### 4.2. Placement Detection with Linear Acceleration and Gravity Information

As mentioned, the linear acceleration and gravity readings provided by the Android API were recorded in the two datasets [11,12]. Following the same methodology in Section 4.1 where features were computed from acceleration readings, the linear acceleration readings are utilized for motion information, whereas orientation information and pitch and roll values are computed from the gravitational readings. Since the random forest classifier achieved the highest scores in the initial set of the experiments, in the rest of the experiments, this classifier is used; hence, the results in this section and the following subsections are achieved with the random forest classifier. In this section, the aim is to investigate how well an accelerometer-only solution performs compared to the use of linear acceleration and gravity readings.

Results of using only linear acceleration readings, gravity values and rotational values using the random forest classifier are presented in Table 6, both in terms of accuracy and the F-measure values, ranging between zero and one (the last row, W-average: weighted average). When the linear acceleration features are used alone, the accuracy is lower compared to the use of both linear acceleration and the gravity features. When we compare these results with the results achieved with motion-related features obtained from raw acceleration readings, presented in Figure 4, these results are lower by 3% for Dataset 1 and by 10% for Dataset 2 on average. Accuracy with raw acceleration was 60% for Dataset 1 and 76% for Dataset 2, whereas it is 57% for Dataset 1 and 65% for Dataset 2 with linear acceleration information.

**Table 6:** Results with linear acceleration and gravity information.

		Linear Acceleration (Motion)		Linear Acceleration and Gravity	
**Dataset**	**Position**	**Accuracy**	**F-Measure**	**Accuracy**	**F-Measure**
Dataset 1 [11]	Messenger bag	52.89	49.04	71.11	68.85
	Pocket	74.03	73.05	88.98	87.24
	Backpack	41.47	44.66	71.81	72.73
	Jacket pocket	59.23	61.27	71.00	73.21
	W-average	57.34	57.35	75.82	75.57
Dataset 2 [12]	Pocket	75.51	75.55	92.88	93.77
	Belt	61.46	60.25	93.83	90.09
	Upper arm	56.93	54.33	90.96	91.98
	Wrist	55.05	58.16	87.28	87.62
	W-average	64.89	64.76	91.57	91.45

When the rotational information and the orientation information from the gravitational readings are added, the accuracies increase for both datasets. However, compared to the results achieved with raw acceleration readings presented in Figure 6, average accuracies are similar, 75% and 91.5%, respectively. For Dataset 2, the results with raw acceleration were only slightly higher by 1.5%. Computing linear acceleration readings generally consumes more battery power and may not be preferred in real-time, continuous-running applications of position recognition.

### 4.3. Placement Detection with the Gyroscope and Magnetometer

Gyroscope and magnetometer readings provided by the Android API were only available in the third dataset [12]. Although the accuracies achieved with this dataset are quite high, around 93%, it is investigated whether the rotational information provided by these sensors may further increase the results. In this set of evaluations, mean, standard deviation, ZCR, RMS and ABSDIFF values for each gyroscope axis are used. In Table 7, the results with only using the gyroscope and also with the accelerometer are presented, both in terms of accuracy and the F-measure. The features computed from acceleration were motion-related features (mean, variance, FFT coefficients, energy, ZCR, RMS and ABSDIFF values for the magnitude) and orientation-related features (standard deviation, ZCR, RMS and ABSDIFF values) obtained from each acceleration axis. When the gyroscope is used alone, accuracies for each of the positions are similar, except the upper arm position. The arm position was mostly confused with the wrist position, since the rotational information was similar.

**Table 7:** Results with the gyroscope and acceleration information.

		Gyroscope		Acceleration and Gyroscope	
	**Position**	**Accuracy**	**F-Measure**	**Accuracy**	**F-Measure**
Dataset 2 [12]	Pocket	89.55	90.15	97.55	97.44
	Belt	88.60	75.32	94.12	93.87
	Upper arm	75.58	89.36	95.23	94.75
	Wrist	84.04	82.32	95.00	95.34
	W-average	85.46	85.46	95.89	95.77

Compared to the results of using acceleration with motion data only given in Figure 4, the gyroscope alone performs better. Again, when orientation and rotation-related information was available from the accelerometer given in Figure 6, using the gyroscope performs better. When the gyroscope readings were coupled with the raw acceleration readings, given in Table 7, accuracies further increase up to 95% on average, whereas this is 98% for the pocket case. This supports the findings of previous work [22,25]. If we compare the results with the results of using all feature information with the accelerometer given in Figure 6 for Dataset 2, on average, the gyroscope and acceleration setting outperforms the only-accelerometer solution by 2.5%. This is due to an 8% increase for the wrist position and 5% for the belt position. Although an accelerometer-only solution provides an efficient solution, the gyroscope can compute exact rotation information, compared to using the accelerometer for computing pitch and roll values. Unfortunately, this cannot be validated with the other two datasets due to missing information.

Another sensor modality available in the second dataset was the magnetic field sensor. In Table 8, results with only using the magnetic field sensor and together with the accelerometer are presented. The magnetic field sensor does not perform as well as the gyroscope alone. Compared to using only motion-related information from the accelerometer given in Figure 4, it performs 8% lower. However, when it is coupled with the accelerometer, it exhibits very similar results to the accelerometer results presented in Figure 6. However, the results of using both the gyroscope and acceleration cannot be reached with the magnetic field and accelerometer readings.

**Table 8:** Results with magnetic field and acceleration information.

		Magnetic Field		Acceleration and Magnetic Field	
	**Position**	**Accuracy**	**F-Measure**	**Accuracy**	**F-Measure**
Dataset 2 [12]	Pocket	70.62	72.00	95.23	95.06
	Belt	77.80	75.34	90.96	92.48
	Upper arm	68.81	64.63	95.84	95.27
	Wrist	49.26	51.67	91.88	90.78
	W-average	67.42	67.13	93.83	93.73

### 4.4. Reduced Number of Positions: Aggregation of Positions

For most applications, it may not be necessary to identify the exact position of the device, but it may be sufficient to know whether the phone is in an enclosed position, such as in a bag, or carried on the upper body, such as attached to the arm. In this section, the performance is investigated by aggregating the available positions to a smaller number of positions. Additionally, when we carefully explore the confusion matrices of the results obtained in Section 4.1, it is observed that the messenger bag position was often confused with the backpack position and that the jacket pocket was often confused with the trouser pocket. These two positions were the positions where the lowest accuracy was achieved. In this set of experiments, only acceleration readings containing motion, orientation and rotation-related information are utilized.

First, the number of positions is reduced to five, as shown in Table 9. In this case, different bag, pocket and hand positions are aggregated to common classes. The results of this experiment are shown in Table 10. The average accuracy increases by 3%, which was 84% when the individual positions were targeted, presented in Figure 7 with the random forest classifier. As mentioned, in the case of exact position experiments, the accuracies of messenger bag and jacket pocket positions were quite low, since they were confused with the other bag and pocket positions. With the aggregation of classes, the bag position is identified with 87% accuracy, whereas the pocket position is correctly classified with 91% accuracy.

Next, the number of classes is reduced to three, bag, pocket and other, as shown in Table 9. In bag and pocket positions, the phones were enclosed, while in other positions, they were not. The enclosed *vs.* not classification was performed in [8], and 85% accuracy was reported. The results with the reduction to these three positions are given in Table 11. The average accuracy is observed to be 88%. Knowing that the phone is in the pocket or in the bag may enable context-aware services, such as preventing pocket dialing, or increasing the volume of an incoming call, or knowing that notifications are not visible to the user [8].

**Table 9:** Reduction of classes.

Original Position	5-Class	3-Class (1)	3-Class (2)	2-Class
Backpack	Bag	Bag	Not attached	Other
Messenger bag	Bag	Bag	Not attached	Other
Trousers pocket	Pocket	Pocket	Lower body	Pocket
Jacket pocket	Pocket	Pocket	Not attached	Pocket
Hand	Hand	Other	Upper body	Other
Belt	Belt	Other	Upper body	Other
Arm	Arm	Other	Upper body	Other
Wrist	Hand	Other	Upper body	Other

**Table 10:** Results of the 5-class experiment.

Position	Accuracy	F-Measure
**Bag**	86.91	84.95
**Pocket**	91.39	86.80
**Arm**	85.60	93.80
**Hand**	93.02	89.10
**Belt**	79.31	82.54
Weighted-Average	87.12	86.87

**Table 11:** Results of the 3-class experiments.

Position	Accuracy	F-Measure	Position	Accuracy	F-Measure
**Bag**	86.30	85.30	**Not attached/ Carried**	92.23	91.41
**Pocket**	85.69	87.88	**Upper body**	94.41	93.27
**Other**	93.76	92.78	**Lower body**	86.57	88.98
Weighted average	88.38	88.67	Weighted average	91.04	91.19

As another three-class problem, I looked at the recognition of whether the phone is attached to the human body or worn/carried by the user. In this set, the classes were upper body, lower body and carried/worn. The aggregation of the original classes to these three positions is presented in Table 9. The results are presented in Table 11. Ninety one percent accuracy is achieved in this set of experiments. Knowing the position of the phone relative to the parts of the body may be useful especially for activity recognition or step counting applications.

As the final evaluation with the reduced number of classes, the in-pocket detection performance is investigated, since this issue has been the subject of research in previous studies [15,16]. Results are presented in Table 12. Although in previous studies, the use of multiple sensors was proposed for in-pocket detection, using only acceleration information also results in a reasonably high accuracy. In [15], the average accuracy was reported as 80% with the use of the microphone with a lower number of other labels.

**Table 12:** Results of the 2-class pocket detection experiments.

Position	Accuracy	F-Measure
**Pocket**	86.09	88.69
**Other**	96.88	95.19
Weighted average	93.31	93.04

The accuracy of pocket position was around 90% for Dataset 1 and Dataset 2 and 83% for Dataset 3 in Figure 6, using motion, rotation and orientation-related features. When datasets are combined, the average accuracy was observed to be 88% in Figure 7. When the number of positions was reduced to five, the pocket position could be recognized with 91% accuracy in Table 10. Hence, quite high accuracy was achieved for the pocket position with the accelerometer, since it can best capture the leg movements with the mobile activities. In the two-class problem, the pocket data were not only from the trouser pocket, but data from the jacket position were also included in this class. This position was often confused with the other pocket position. One of the reasons for the higher accuracy is the combination of these data. Another reason could be due to the higher number of instances of the pocket position in the combined dataset, since it is the common position in all datasets. It would be interesting to show the average accuracy, when the dataset is class balanced, for this experiment.

### 4.5. Impact of Activity Types

In previous evaluations, all of the position data was collected with different activities, such as walking, running and sitting. In this section, it is investigated how much the activity information impacts the phone position identification. For this purpose, first, I process the data when the participants were only walking. Position identification with the walking activity was investigated in previous studies [19,21]. In [19], accuracy was reported as 94% with bag, ear, hand and pocket positions. In [21], using the SVM classifier, 91% accuracy was achieved with four phone positions, utilizing the cross-validation approach. Walking activity exhibits a periodic behavior, and hence, it is easier to recognize the positions. For a practical application, however, first, it should be identified whether the user is walking. Although this can be performed with a reasonable accuracy, it may not be 100%, and this may reduce the position identification accuracy.

**Figure 8:**
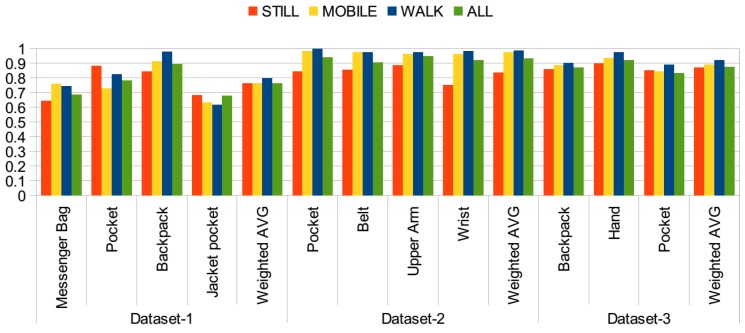
Accuracy when the activity information is available, individual datasets.

**Figure 9:**
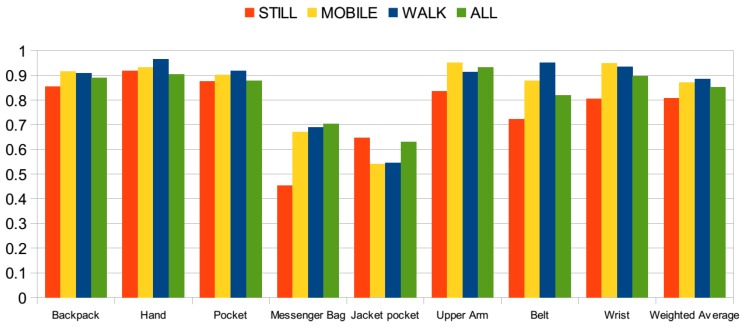
Accuracy when the activity information is available, datasets combined.

The results of this evaluation are presented in Figure 8, where evaluations on individual sets are carried and in Figure 9, where the datasets are combined into a single dataset. The *y* axis represents the accuracy values in both figures. In this set of evaluations, only acceleration readings containing motion, orientation and rotation-related information are used. In both of the figures, the highest accuracies are often achieved with the walking activity. When individual datasets are considered, in Figure 8, the accuracy achieved with the second dataset with the walking activity is very high, close to 100% accuracy. Similarly, in the other two datasets, higher accuracies are achieved compared to the use of data including all activity information. However, for pocket positions, including jacket pocket, accuracies are lower with the walking activity compared to stationary, *i.e.*, still, activities. This is due to the fact that the signal behavior is very similar in these positions. In the case of walking activity, the jacket pocket was mostly confused with the trouser pocket, backpack and messenger bag positions.

When the datasets are aggregated into a single dataset in Figure 9, accuracies for the hand, pocket, messenger-bag and belt positions are the highest with the walking activity. However, in the jacket pocket position, lower accuracy is observed, since it is confused with the pocket, backpack and messenger bag positions, as shown in the confusion matrices given in Table 13. In the jacket position, the phone is placed close to the waist and hip and experiences a similar motion pattern as the pocket position and messenger bag positions. In the backpack position, also the accuracy with the walking activity is quite high, but it increases almost by 1% with mobile activities.

Next, the performance with stationary activities is evaluated. Although it may be challenging to detect a position with an accelerometer when there is no motion and rotation-related information, the orientation of the phone provides useful information about its position. In the two datasets [11,12], all phones were stationary during standing and sitting activities, whereas in one dataset [13], users were making phone calls, sending an SMS and interacting with the phone positioned in the hand. Hence, this makes it more challenging, but more realistic, since users interact with the phone in these states more than the other activities. Results are presented again in Figure 8 for individual datasets and in Figure 9 for the combined dataset. In Dataset 1, the average accuracy is higher compared to the use of all activities, since the messenger bag and jacket pocket positions are identified with a higher accuracy. In Dataset 2, however, the average accuracy drops to 83%, since it includes positions that can be identified better with motion information. In Dataset 3, similar average accuracy to that with all of the activity information is achieved. Although the accuracy for the backpack position decreases, it increases for the pocket position. For the combined dataset, Figure 9, the performance is similar in terms of average accuracy. The confusion matrices obtained with stationary activities are presented in Table 14. Compared to the walking activity, in all of the positions (except the jacket pocket), a decrease in accuracy is observed, ranging from 4% in the pocket position to 22% in the belt position. However, in the jacket pocket position, the accuracy increases by 10%. It is again confused with the messenger bag and pocket positions, but with a lower rate of confusion with the backpack position. In the hand position, a quite high accuracy is observed, since users were interacting with the phone during still activities. The belt position is confused with the pocket position, which is expected due to similar orientations during the stationary activities. Similarly, the wrist position is confused with pocket.

**Table 13:** Confusion matrices with the walking activity, random forest, in %.

	Backpack	Hand	Pocket	Messenger Bag	Jacket Pocket	Arm	Belt	Wrist
Backpack	**90.92**	7.09	0.77	0.07	1.14	0	0	0
Hand	3.13	**96.61**	0.24	0	0	0	0	0.03
Pocket	4.58	0.35	**91.79**	2.29	0.99	0	0	0.01
Messenger bag	8.40	0.06	4.91	**68.98**	17.66	0	0	0
Jacket pocket	14.83	0.07	18.32	12.17	**54.61**	0	0	0
Arm	0	0	0.68	0	0	**91.36**	0	7.95
Belt	0	0	4.77	0.11	0	0	**95.11**	0
Wrist	0.11	0.57	5.34	0	0.11	0.34	0	**93.52**

**Table 14:** Confusion matrices with stationary activities, random forest, in %.

	Backpack	Hand	Pocket	Messenger Bag	Jacket Pocket	Arm	Belt	Wrist
Backpack	**84.82**	6.80	2.30	3.20	2.82	0.08	0.00	0.00
Hand	5.66	**91.92**	2.11	0.02	0.30	0.00	0.00	0.00
Pocket	2.40	3.87	**87.59**	2.43	0.10	0.48	1.21	1.92
Messenger bag	18.58	0.09	21.38	**45.45**	12.82	0.00	1.69	0.00
Jacket pocket	2.72	0.17	6.16	26.09	**64.86**	0.00	0.00	0.00
Arm	0.40	0.23	2.54	0.00	0.00	**83.67**	0.45	12.71
Belt	0.11	0.40	15.42	0.00	0.06	5.03	**72.32**	6.67
Wrist	0.17	0.00	17.12	0.00	0.00	1.19	1.02	**80.51**

Finally, the performance when subjects are mobile, *i.e.*, walking, jogging, running, biking, going up/down stairs and on a bus, is evaluated. The results of this set are presented again in Figure 8 for individual datasets and in Figure 9 for the aggregated dataset. Similar to the results achieved with the walking activity, average accuracies for individual datasets are higher with the mobile activities, compared to the results with all activities. Again, the messenger bag and the jacket pocket are the lowest scoring positions, particularly in the individual datasets, as explained for the walking activity. The messenger bag position is often confused with the jacket pocket and backpack positions, whereas the jacket pocket is often confused with the messenger bag, pocket and backpack positions. The confusion matrices for the mobile activities are presented in Table 15. Compared to the walking activity, in the upper arm and wrist positions, a higher accuracy is observed with the mobile activities, by almost 4% and 1.3%, respectively. Arm position was confused with the wrist during walking, and the wrist position was confused with the pocket.

**Table 15:** Confusion matrices with the mobile activities, random forest, in %.

	Backpack	Hand	Pocket	Messenger Bag	Jacket Pocket	Arm	Belt	Wrist
Backpack	**91.70**	5.02	2.25	0.99	0.05	0.00	0.00	0.00
Hand	4.82	**93.29**	1.89	0.00	0.00	0.00	0.00	0.00
Pocket	5.21	0.97	**90.21**	1.17	1.56	0.51	0.11	0.25
Messenger bag	8.97	0.26	6.61	**67.10**	13.76	0.00	3.25	0.04
Jacket pocket	11.48	0.50	12.86	15.55	**53.99**	0.55	4.90	0.17
Arm	0.02	0.00	2.10	0.00	0.00	**95.20**	0.00	2.69
Belt	0.00	0.00	9.08	2.00	0.51	0.48	**87.89**	0.05
Wrist	0.07	0.07	2.71	0.06	0.02	1.68	0.53	**94.85**

## 5. Discussion

In this section, the findings obtained in Section 4 are elaborated, and a more in-depth discussion on the results is provided with insights from this work, as well as comparisons with related studies utilizing an acceleration-only solution.

Number of classes and similarities among classes are important, use large datasets: First of all, as in other classification problems, the number of classes and the similarities among the classes impact the results. Comparing the results when datasets are analyzed individually using all features, given in Figure 6, and when they are combined, given in Figure 7 (MOR column), in most of the positions, accuracies either stay the same or decrease when the datasets are combined. The maximum decrease is for the belt position of Dataset 2 [12], which is around 8%. When this dataset was analyzed individually, the belt position was often confused with the pocket position, as seen in Table 3. However, when the datasets are combined, there are more instances of pocket positions coming from Dataset 1 and Dataset 3, and the confusion with this position has increased. If we look at the pocket position available in all three sets, when the datasets are combined, the accuracy increase for the pocket position of Dataset 1 [11] is around 10% higher (from 78% to 88%), whereas it is 5% higher (from 83% to 88%) for the pocket position of Dataset 3 [13] compared to the results of individual datasets. The accuracy of pocket position for Dataset 2 [12] was around 94% when this dataset was analyzed individually, and it decreased to 88% when datasets are combined. In this dataset, the other classes were easier to differentiate, such as arm, wrist and belt, since they are related to different parts of the body. As a summary, although high accuracies can be reported with a lower number of positions, for a realistic comparison, the most common positions identified in surveys should be considered [8].

Difficult to detect bag positions: The bag positions are difficult to recognize. In the second dataset, there is no bag position, whereas in the third dataset, only the backpack position is available, and the accuracies of these positions are higher. Additionally, the acceleration readings in the jacket pocket position are also similar to bag positions and trouser pocket positions; hence, it is a difficult position to identify with the accelerometer. When datasets are combined, the accuracies for the messenger bag and jacket pocket are relatively poor compared to the cases where Dataset 1 was analyzed individually. In the combined datasets, the number of classes is higher, and since usually the messenger bag is confused with the backpack position and the jacket position with the trouser pocket position, with a higher number of instances in the combined dataset, the accuracies of these two positions are relatively low. The same findings for the jacket pocket were reported in [20], and a lower rate of recognition of the bag position was reported in [8]. However, if knowing the exact position is not necessary and different bag and pocket positions can be combined, the messenger bag and jacket positions can also be recognized with a higher accuracy as bag and pocket positions as investigated in Section 4.4.

Fuse motion information with orientation and rotation: When motion, orientation and rotation-related information is used separately for position recognition, motion-related features are observed to be more dominant compared to others. However, they alone do not perform well, and when they are combined, especially with rotation-related features, the accuracies significantly increase. The combination of orientation and rotation information without the motion information does not exhibit high levels of accuracy. Since in most of the activities performed in all three sets, the phones were mobile, it was expected that motion information dominates, but in stationary activities, orientation and rotation-related information can be more useful. This can be investigated in future work.

Rotation information is useful at positions where the phone is not tightly placed: The use of rotation features was only discussed in [21]. However, the gyroscope was used for extracting rotation information. In this paper, I investigated how rotation-related information can be extracted from the accelerometer. It is shown that rotation-related features are especially useful in identifying positions where the phone is not tightly placed, such as the jacket pocket and messenger bag positions, compared to other positions where the rotations are not dominant, such as the belt.

The acceleration-only solution performs well, and it is not necessary to use other motion-related sensing modalities: Instead of using only acceleration information, other sensing modalities related to motion and rotation can also be utilized for position recognition. For instance, using linear acceleration and gravity readings performed similarly to using only acceleration information. However, computing linear acceleration readings generally consume more battery power and may not be preferred in real-time, continuous-running applications of position recognition. Although an accelerometer-only solution provides an efficient solution, the gyroscope can compute exact rotation information, compared to using the accelerometer for computing pitch and roll values. As shown in Table 7, using the gyroscope besides acceleration improved the results by 3% for Dataset 2 [12]. Unfortunately, this cannot be validated with the other two datasets due to missing information, and in future work, this can be considered in detail. Another sensing modality available in Dataset 2 was the magnetic field. Similar to the gyroscope, it can be used to extract rotation information. However, it did not perform as well as the gyroscope, and the results were similar to the acceleration-only solution.

Merge positions if exact placement identification is not necessary: Comparing to exact position identification with in-pocket detection, on-body detection and bag pocket detection problems, higher accuracies can be achieved since similar positions are aggregated into the same class. In the two-class problem, the pocket data are not only from the trouser pocket, but data from the jacket position are also included in this class. This position was often confused with the other pocket position. One of the reasons for the higher accuracy is the aggregation of these two positions.

Walking makes recognition easier, but even with stationary activities, acceleration-only solutions perform well: In the utilized datasets, subjects were performing different activities while carrying the phones at different positions. These include mobile activities, such as walking, running, transportation in a bus, stairs and biking, and stationary activities, such as sitting and standing. When we investigate the impact of the activity on position recognition performance, it is observed that for most of the positions, the highest accuracy is achieved with the walking activity due to periodic movement. However, the jacket pocket is identified with a higher accuracy with stationary activities, utilizing the orientation information. On the other hand, with other positions, such as the belt, wrist and upper arm, lower accuracies were observed with stationary activities. If position recognition is provided as a service running on the phone, it would be efficient first to detect whether the phone is stationary or mobile, and then, the position recognition can be performed, as also discussed in [18,22]. To the best of my knowledge, phone position identification with only stationary activities using an accelerometer-only solution was not investigated in previous studies. I explore the impact of the performed activities on position recognition and show that the accelerometer-only solution can achieve 80% recognition accuracy with stationary activities where movement data are very limited.

Comparison with other acceleration-only solutions: A comparison of this work with the recent findings in the literature utilizing an accelerometer-based placement recognition method is presented in Table 16. Except [9,18], the other studies presented in the table use a smart phone integrated accelerometer, whereas in [19], the Nokia Sensorbox was connected to a Nokia phone via Bluetooth connection. In [9,18], wearable accelerometers were used. In some of the studies, a cross-validation approach was used; however, I test the classifiers with the leave-one-subject-out validation strategy (LOSO), since the aim is to explore how the placement recognition can be generalized to new subjects without the requirement of user-specific training data. Compared to the studies that use the LOSO approach [8,18,20], it is shown that a higher recognition accuracy can be achieved, except [18]. However, the number of placement sites in my study is larger than the number of locations utilized in [18], and different parts of the body were targeted in that study. With respect to the previous studies, in this study, classifiers were tested on a much larger dataset, involving more participants and activities. The only exception was Mannini *et al.*’s work [18], where they also work on a complex dataset. However, as mentioned, the target positions are different. If we compare the results that we achieve with Dataset 2 [12], where similar positions are targeted (pocket, belt, upper arm, wrist), with their findings, 93% accuracy was achieved when all activity data was used (Figure 6), and 98% accuracy was achieved when only walking data are used; see Figure 8. As future work, it would be interesting to test their method (first walking detected) on my dataset.

**Table 16:** Comparison with other accelerometer-based phone placement solutions. LOSO, leave-one-subject-out.

	Park *et al.* [19]	Wiese *et al.* [8]	Fujinami *et al.* [20]	Alanezi *et al.* [22]	Wahl *et al.* [25]	Kunze *et al.* [9]	Mannini *et al.* [18]	This Work
**Positions**	4: Bag, ear, hand, pocket	4: Pocket, bag, hand, out	9: Neck, chest, jacket pocket, trousers pockets (front, back), backpack, handbag, messenger bag, shoulder bag	6: hand-holding, talking on phone, watching a video, pockets (pants, hip, jacket)	5: Pants, table, jacket, bag, no label	5: Head, wrist, torso pockets (front, back); AND 5: hand, wrist upper arm, knee and back	5: Ankle, thigh, hip, upper arm, wrist	8: Backpack, hand, pocket, messenger-bag, jacket-pocket, arm, belt, wrist
**Activities**	Walking	sitting (on a couch, on a desk chair), standing, walking	Walking	Idle, walking, running	Working, eating, walking/cycling, Vehicle	activities of daily living, household, workshop and office activities	Lying (on back, on left side, on right side), sitting (Internet searching, typing, writing, reading), standing still, sorting files on paperwork, exercise bike, cycling (outdoor level, outdoor uphill, outdoor downhill), elevator (up, down), jumping jacks, sweeping with broom, painting with roller, painting with brush, walking, stairs	Sit, stand, bike, walk, run, jog, stairs (up/down), transport (bus), secondary activities: sending an SMS, making a call, interaction with an app
**No. of Subjects**	14	15–32	20	10	6	17	33	35
**Features**	Frequency domain	Time and frequency domain	Time and frequency domain	Time domain	Time domain	Time domain	Time and frequency domain	Time and frequency domain
**Classifier**	C4.5, SVM	SVM, random forest	J48, SVM, naive Bayes, MLP	J48, naive Bayes, logistic regression, MLP	Nearest centroid classifier	HMM + particle filter smoothing	SVM	J48, KNN, random forest, MLP
**Validation method**	10-fold cross	LOSO	LOSO	10-fold cross	10-fold cross	Train/test over randomly-picked subset	LOSO	LOSO
**Recognition accuracy**	99.6%	79% (random forest)	74.6%	88.5% (only accelerometer, first activity detected, J48)	82% (only accelerometer)	82.0%	92.7% (first walking activity detected)	85% (datasets combined)
**Recognition accuracy, walking**	99.6%	Not reported	74.6%	Not reported	Not reported	94.9%	81%	88% (datasets combined)

Although in this work, the focus was on an accelerometer-only solution and acceptable accuracies are achieved, the accelerometer can be coupled with other sensing modalities if higher accuracies are targeted. However, the requirement should be that, if phone placement recognition is provided as a service on a phone, it should not consume much battery power. In this respect, using new techniques, such as RFID tags [24,25], can be considered.

## 6. Conclusions and Future Work

In this paper, the focus was on the phone placement/position recognition problem, and the use of an acceleration-only solution is investigated with a comprehensive analysis. The investigation is performed on a relatively large and complex dataset, which is a combination of three different datasets, including 35 subjects, eight phone positions with eight different types of activities performed by participants. Initially, it is investigated whether the phone position can be detected efficiently by analyzing the movement, orientation and rotation changes. Following a systematic approach, the use of motion, orientation and rotation-related features is explored where different combinations of feature sets are considered. It is shown that using only motion, orientation or rotation information performs poorly; however, when features are fused, the average accuracy increases by 15% for the combined dataset, resulting on average in 85% accuracy, where the random forest classifier achieved the highest accuracy. Next, it is investigated whether an accelerometer-only solution performs as well as utilizing other sensing information, such as linear acceleration, gravity, gyroscope and magnetic field sensors. It is observed that these sensors alone cannot perform as well as the accelerometer. However, particularly when the gyroscope is used with the accelerometer, a 2% to 3% increase in accuracies is observed compared to the use of the accelerometer-only approach. Other position detection problems, such as in-pocket *vs.* not, on upper/lower body *vs.* not, bag pocket *vs.* other classes, are also studied. Eighty eight percent accuracy for three-class problems and 93% accuracy for in-pocket detection were observed with the random forest classifier, using only the accelerometer. Finally, the impact of the activities performed by the users during position detection is analyzed, particularly focusing on walking, stationary and mobile activities. It is shown that the walking activity makes it easier to recognize positions due to its periodicity in most of the positions. Additionally, it is shown that an accelerometer-only solution can achieve 80% recognition accuracy even with stationary activities where movement data are very limited.

As future work, I plan to generalize my findings on other datasets and aim to create a pool of datasets for phone position recognition to cover the missing positions in the utilized datasets. For example, on table/desk/shelf positions are common positions when the user is not interacting with the phone, and they may be confused with other positions when the user is stationary. It would also be interesting to analyze transition states, such as identifying when a phone in the pocket is taken out to answer an incoming call. The use of the findings on a practical position recognition application running on a phone all day long will be also investigated, and its performance will be tested in relation to a context-aware application.
